# Effects of physical activity and self-control on mobile phone addiction in college students: a cross-lagged study in China

**DOI:** 10.3389/fpsyg.2024.1417379

**Published:** 2024-07-19

**Authors:** Qinghe Wang, Yanke Chen, Lan Li

**Affiliations:** ^1^Department of Physical Education, Kyonggi University, Suwon-si, Republic of Korea; ^2^School of Physical Education, Zhengzhou Normal University, Zhengzhou, China

**Keywords:** physical activity, self-regulation, college students, smartphone addiction (MPA), longitudinal surveys

## Abstract

**Purpose:**

This study aims to investigate the impact of physical activity and self-control on college students' mobile phone addiction through cross-lagged longitudinal surveys, addressing the limitations of previous cross-sectional studies.

**Patients and methods:**

A total of 414 college students were tracked three times during a 12-month period using the Physical Activity Rating Scale-3 (PARS-3), the Mobile Phone Addiction Tendency Scale (MPATS), and the Brief Self-Control Scale (BSCS). AMOS25.0 software was used to construct the cross-lagged relationship model, and the maximum likelihood approach was employed to investigate the model fitting. The asynchronous correlation between variables was investigated from the time series through the cross-lagged path coefficient.

**Results:**

The fitting indexes of the cross-lagged model showed *x*^2^/df = 5.098, GFI = 0.977, NFI = 0.969, IFI = 0.975, CFI = 0.974; RMSEA = 0.100, and SRMR = 0.030. The calculation conducted by combining the path coefficient of the model shows that PA and SC are the antecedent variables of MPA, and PA is the antecedent variable of SC. In addition, SC serves as a mediator in the path of PA, affecting MPA.

**Conclusion:**

(1) physical activity can positively affect subsequent self-control; (2) physical activity can negatively influence subsequent mobile phone addiction; (3) self-control can negatively affect subsequent mobile phone addiction; and (4) physical activity can indirectly influence subsequent mobile phone addiction through self-control.

## 1 Introduction

The widespread use of smartphones has revolutionized our daily lives, bringing unparalleled convenience and connectivity. However, this technological advancement has also led to significant social issues, such as mobile phone addiction (MPA). Mobile phone addiction refers to the physical or psychological discomfort resulting from the improper use of smartphones, which can affect individual mental health and social functioning (Walsh et al., 2010). Due to the portability of mobile phones, mobile phone addiction is more pervasive, hidden, and harmful compared to traditional computer or Internet addiction. Psychiatrists predict that mobile phone addiction will be one of the most critical forms of non-drug dependence in the twenty-first century (Shambare et al., 2012). According to previous studies, MPA can lead to sleep disorders, depression, and anxiety in college students and damage their physical and mental health (Thomée et al., 2011; Demirci et al., 2015; Elhai et al., 2016; Enez Darcin et al., 2016).

In view of the serious harm of smartphone addiction, effective interventions for smartphone addiction have been proposed in recent years, such as group counseling, mindfulness intervention proposed by Lan et al. (2018), Garland and Howard (2018), and Sancho et al. (2018) meditation proposed by Choi et al. (2020) and cognitive behavioral therapy proposed by Kim et al. (2012), Kim et al. (2018), Zhang et al. (2020), and Singh and Samantaray (2022). At present, scholars have verified the effectiveness of behavioral therapy, including reality therapy, cognitive behavioral therapy, positive psychological intervention, and family-based group therapy, for MPA. Nevertheless, some other scholars have also explored the effects of adjuvant therapy, such as exercise or physical activity (Kim, 2013). Besides, the positive impact of physical activity on alleviating MPA has even been validated in some research (Fan et al., 2021; Yang et al., 2021). Besides, a large body of evidence has shown that individuals lacking self-control are more prone to MPA (Jiang and Zhao, 2016; Cho et al., 2017; Yang et al., 2019).

In recent years, some scholars have used cross-sectional research design to explore the association between three terms, i.e., physical activity, self-control, and MPA. A series of studies have shown that self-control acts as an intermediary variable between physical activity and MPA (Zhong et al., 2021; Zhang et al., 2022) or a moderating variable (Xiang et al., 2020; Zeng et al., 2022). However, previous researchers mostly used a cross-sectional research design to study the relationship between physical activities and the other two variables. The research results mostly focused on the one-way relationship between variables, and few researchers paid attention to the interaction mechanism between these three variables across time points. At the same time, scholars also believe that the cross-sectional design cannot provide evidence for the causal relationship between variables (Xiang et al., 2020; Zeng et al., 2022).

To compensate for the shortcomings of cross-sectional research, this study adopted the longitudinal research design of a cross-lagged path model and selected 414 college students from an undergraduate college in Hunan Province as the research object. After three surveys on these students in the 12-month observation period (with a survey interval of 6 months), this study explored the possible associations between physical activity, self-control, and MPA.

Our contribution to academics has two aspects. First, this study verifies the robustness of physical activity in alleviating the MPA of college students and further examines its potential mechanism. Second, this study investigates the longitudinal predictive association between physical activity, self-control, and MPA, which can provide a reference for subsequent research.

The structural layout of the article is as follows: The first part is the literature review on physical activity, self-control, and mobile phone addiction. Using this literature, we propose possible relationships between variables, such as physical activity, which can reduce mobile phone addiction, and physical activity, which can positively affect self-control. Then, we propose our research methods, including our data, variables, and cross-lagged models. The final part is our results and discussions of our findings, highlighting the significance and limitations of this study and the recommended study methods for future research.

## 2 Literature review

### 2.1 Mobile phone addiction

Griffiths was the first to define MPA. He classified MPA as a technical-level addiction, which is an addictive behavior stemming from people's increasing attachment to mobile phones in their daily interactions (Griffiths, 1995). Park defines MPA as an addiction that arises from habitual reliance on mobile phones. He underscores the adverse consequences of this dependency, emphasizing its negative impact on people's lives (Park, 2005). According to Yen et al., MPA is an addition to diverse activities related to mobile phones, characterized by immersion and a heavy reliance that significantly affects individuals' psychological health and social functions (Yen et al., 2009). Vacaru et al. defined MPA as any problem arising from the use of mobile phones, encompassing three dimensions: physical, social, and psychological (Vacaru et al., 2014). Choliz provides five dimensions to define MPA: excessive use manifested as high economic costs and a large number of phone calls and short messages; related problems caused by the excessive use of mobile phones, such as conflicts with parents; interference with school and personal activities; gradually increasing use to achieve the same satisfaction; a demand for replacing phones frequently; and emotional fluctuations caused by inaccessibility to mobile phones (Choliz, 2012).

Additionally, Oniz et al. discussed the broader impact of MPA, highlighting the negative consequences, such as increased stress and anxiety levels, and the impact on physical health, including obesity (Oniz et al., 2023). Gocer and Oniz further elaborated on the psychological and social dysfunctions caused by MPA, providing a comprehensive framework for understanding its impacts on young athletes, including distraction, sleep problems, and reduced athletic performance (Gocer, 2023).

The definition of MPA by peers mainly emphasizes the long-term overuse of mobile phones and the resultant damage and adverse effects on physical, psychological, and social functions. Therefore, this study defines MPA as an obsession that leads to impaired psychological and social functions due to long-term overuse of mobile phones driven by a certain motivation.

### 2.2 Physical activity

Physical activity refers to sports activities that people choose according to the body's physiological and psychological needs. These activities combine different sports methods to develop physical fitness, improve physical and mental health, enhance physical fitness, and enrich cultural life. Generally speaking, physical activities include the following elements:

- Frequency refers to the number of sports activities in a period of time.
- Duration refers to the time spent on an exercise activity.
- Intensity refers to the load of participating in sports activities and the degree of physiological strength.

Research has shown that physical activity can significantly reduce stress levels and improve overall wellbeing. According to a study by Emlek et al., regular engagement in physical activity is associated with lower levels of anxiety and depression among adolescents. This study highlights the importance of incorporating physical activity into daily routines to combat mental health issues related to sedentary behavior and MPA (Emlek et al., 2023).

Furthermore, a study by Ugurlu et al. emphasizes the role of physical activity in enhancing cognitive functions, such as memory and attention, which are often impaired in individuals with high levels of MPA. The findings suggest that physical activity can serve as a protective factor against the negative cognitive impacts of excessive mobile phone use (Ugurlu et al., 2023).

### 2.3 Self-control

Kopp pointed out that self-control is an individual's ability to control their behaviors independently and promote their personal values to match social expectations. It can trigger or stop certain behaviors, such as inhibiting impulses, resisting temptations, delaying gratification, formulating and completing behavioral plans, and adopting behavioral patterns that match social situations (Kopp, 1982). Baumeister et al. consider self-control as a process in which individuals monitor and suppress certain inherent behavioral tendencies and align individual behavior with self-worth or social norms (Baumeister et al., 1994). Telzer et al. argued that self-control is the ability to regulate, manipulate, and control one's thoughts, feelings, and behaviors related to impulsivity (Telzer et al., 2011). Sjastad et al. defined self-control as individuals' ability to regulate responses to overcome impulses to achieve a person's goals or achieve a person's value (Sjåstad and Baumeister, 2018).

To sum up, based on the common ground of the above literature, this study finally defines self-control as individuals' ability to consciously restrain and regulate their cognition, emotion, and behavior in accordance with social expectations and requirements to achieve their long-term goals without external supervision and restrictions.

## 3 Physical activity, self-control, and mobile phone addiction

### 3.1 Physical activities and mobile phone addiction

Physical activity plays a crucial role in maintaining individual health and managing and preventing the human body from developing behavioral addiction or mental illness (Marconcin et al., 2022). Guo et al. found a negative prediction impact of physical activity on MPA by investigating 1433 Chinese college students (Guo et al., 2022). The most obvious manifestation of the dose-dependent association between physical activity and MPA appears when individuals in a sedentary state start to do moderate physical activity (Lian et al., 2021). Xiao et al. conducted an intervention test by requiring participants to take on basketball and Baduanjin activities for 12 weeks, finding that physical activity significantly alleviated MPA among college students (Xiao et al., 2021). In the psychological field, Morgan proposed the “distraction theory,” postulating that shifting attention from unpleasant stimuli or painful physical complaints can lead to post-activity emotional improvement (Morgan, 1985). According to the famous self-efficacy theory and mastery hypothesis, physical activity empowers individuals with a sense of refreshing and achievement after exercise (Marcus, 1995; Paluska and Schwenk, 2000). In the Interaction of Person-Affect-Cognition-Execution (I-PACE) model proposed by Brand et al., addictive behavior is mainly characterized by affect and cognitive reactions (Brand et al., 2019). Thus, individuals who engage in moderate physical activity will not spend excessive time using mobile phones (Tao et al., 2020). In addition, Grasdalsmoen et al. proposed that exercise can help individuals cope with emotional sickness associated with mobile phone addiction, including pressure, anxiety, and depression (Grasdalsmoen et al., 2020). Besides, Fan et al. claimed that moderate-intensity exercise enhances the inhibitory control of smartphone-addicted college students (Fan et al., 2021).

### 3.2 Physical activity and self-control

The self-control strength model likens self-control to muscles. Just as muscles can become stronger with regular exercise, self-control can also improve through practice and effort (Yang et al., 2019). As a low-cost, easy-to-carry, and non-side-effect effective means of self-control improvement, physical activity has been confirmed by relevant research: a horizontal study validates that the physical exercise volume can improve people's self-control ability (Schöndube et al., 2017). and a longitudinal study observed the positive effect of chronic and acute physical activity on the self-control ability (Benzing et al., 2018). In addition, Smith et al. and Dwyer et al. claimed that physical exercise can also benefit the human brains' cognition control system (Smith et al., 2010; Dwyer et al., 2014). Some scholars have also observed the different impacts of physical exercise on self-control abilities caused by varied intensity. Fan et al. pointed out that moderate physical activity can improve college students' self-control and ability to avoid being addicted to mobile phones (Fan et al., 2021). However, some scholars have pointed out that high-intensity physical activity may not enhance individual self-control (Kamijo et al., 2007). Davis et al. believed that group activities such as football or basketball can also effectively improve self-control (Davis et al., 2011).

### 3.3 Self-control and mobile phone addiction

The dual-systems perspective of impulse and self-control shows that an imbalance between the impulse and self-control systems significantly contributes to problem behavior associated with low self-control (Hofmann et al., 2009). In other words, low self-control hampers an individual's ability to manage impulses, making them more prone to seeking instant gratification. This lack of self-control makes it difficult to resist the temptations presented by mobile phone content, functions, and games, increasing the likelihood of excessive use of mobile phones.

According to Brand et al.'s I-PACE theory, inadequate control over one's executive and inhibitory abilities diminishes the motivation needed for effective inhibition, leading to additive behaviors (Brand et al., 2016). People with weak self-control struggle to resist habitual behavior, such as using mobile phones. They are more inclined to seek immediate pleasure, current desires, novel experiences, and so on. Poor planning skills, low response inhibition, and the absence of mobile phones often lead to feelings of loss and boredom (Zhong et al., 2021).

Previous research has revealed a significant relationship between self-control and all dimensions of mobile phone addiction. Scholars have found that individuals with lower self-control abilities are more likely to exhibit high levels of MPA (Jiang and Zhao, 2016; Cho et al., 2017; Yang et al., 2019).

### 3.4 The relationship between physical activity, self-control, and MPA

The association between physical activity, self-control, and MPA has attracted great attention. Scholars have found a negative correlation between the former two terms, physical activity and self-control, and the term MPA. Besides, physical activity and self-control can significantly predict MPA. Engaging in physical activity can reduce MPA to some extent, with self-control playing an important mediating role (Yang et al., 2019). A study involving 1,801 Chinese college students found that self-control acts as a mediator in the relationship between physical activity, subjective wellbeing, and MPA (Ding et al., 2021). A South Korean study showed that physical activity and attention control influence MPA levels (Choi, 2015). According to cross-sectional research, there is a negative correlation between physical activity and MPA. Physical activity plays a positive role in self-control, and self-control, subsequently mediates the impact of physical activity on MPA (Zhong et al., 2021; Zeng et al., 2022; Zhang et al., 2022). In addition, MPA may increase sedentary behavior and divert attention from physical activity, while self-control helps mitigate the relationship between sedentary behavior and MPA, thereby promoting a more active lifestyle (Barkley and Lepp, 2016; Xiang et al., 2020).

### 3.5 Cross-lagged panel model

The cross-lagged panel model is a statistical analysis method that utilizes two or more sets of panel data within a longitudinal cohort to investigate the temporal relationships between two interrelated variables. This method addresses the limitations of cross-lagged correlation by incorporating autocorrelation coefficients and considering the time stability of variables in the model (Reed and Verran, 1988). Furthermore, by comparing the cross-lagged path coefficients, a conclusion about the time series relationship between variables can be drawn (Hamaker et al., 2015). Compared to analysis models based on cross-sectional design, the cross-lagged model offers the following main advantages: First, the cross-lagged model examines causality over time, allowing for more detailed and accurate inferences about causal relationships and reducing parameter bias that may occur with cross-sectional data (Selig and Preacher, 2009). This ensures that the cause precedes the effect, fitting the premise condition of the causal effect. Second, the traditional cross-lagged model controls for the stability of variables through the autoregressive effect. Past behavior is a powerful predictor of current behavior, and including previous measurement values as control variables prevents biased cross-lagged effects and erroneous causal inferences. Third, the cross-lagged model can be used to compare the absolute value of the standardized coefficient of the two-way cross-lagged effect so it can determine the strength of the causal relationship between variables. In addition, if the data contain self-reported information, the common method bias caused by the respondent's personality and response styles can significantly impact the accuracy and effectiveness of the results (MacKenzie and Podsakoff, 2012). In this regard, the cross-lagged path model effectively alleviates the negative impact of the common method bias by conducting measurements at multiple time points, thus making the model estimation and fitting results more reliable. In view of this, this study uses the cross-lag path model research design to study the relationship between physical activity, self-control, and MPA.

## 4 Materials and methods

### 4.1 Participants and procedures

In this research, we used a convenient sampling method to select sophomore students from an undergraduate college in Xiangtan City, Hunan Province, China, as the research subjects and conducted a 12-month, three-stage longitudinal follow-up survey. The first test (T1) was conducted from March 10–14, 2022, yielding 528 valid subjects. The second test (T2), conducted from September 11–15, 2022, resulted in 461 valid subjects, resulting in a 12.7% loss rate compared to the first test. The third test (T3) was conducted from March 10–14, 2023, and included 414 valid subjects, reflecting a 10.2% loss rate compared to the second test. There were 197 male students and 217 female students in the effective subjects. The average age was 20.60 ± 0.83 years. In addition, after analysis and testing, the difference between the analysis sample and the loss sample across the three variables is not significant (*P* > 0.05), indicating a non-structural loss.

Three groups of tests were conducted after we obtained informed consent from the participant students and the university. The questionnaires were filled out at the agreed-upon after-school time and collected immediately. The contents and procedures of the three tests were essentially the same, with only the order of some questions adjusted. During each test, 1–2 school principals were present. The principal's role was to explain the instructions to the subjects, outline the purpose and significance of the test, and emphasize the principles of confidentiality, the fact that there were no right or wrong answers, and the need for independent responses. They also explained the sample questions, addressed any potential issues, and monitored the quality of the testing process. The main experimenters were graduate students majoring in psychology who had undergone unified training. Each participant had 15 min to fill out the questionnaires.

## 5 Measures

### 5.1 Physical Activity Rating Scale-3

This study employed the Physical Activity Rating Scale-3 (PARS-3), which was revised by Chinese scholar Liang Deqing in 1994 based on the initial version proposed by Japanese scholar Hashimoto in 1990 (Liang, 1994). According to this scale, the level of physical activity is evaluated in three dimensions: exercise intensity, exercise time, and exercise frequency. The exercise time is graded from 0 to 4, while the exercise intensity and frequency are graded from 1 to 5. The level of physical activity is calculated using the formula: physical activity = intensity × (time-1) × frequency. A higher score indicates a higher level of physical activity, indicating that the individual engages in a greater amount of physical exercise. The Cronbach's α of the physical activity scale in this study was 0.846 (T1), 0.822 (T2), and 0.832 (T3), respectively.

### 5.2 Brief Self-Control Scale

In 2004, Tangney et al. published the Self-Control Scale (SCS), which has become the most widely used scale worldwide for calculating self-control levels (Tangney et al., 2004). Although the scale can comprehensively evaluate self-control, there are still some limitations, such as being time consuming, prone to response fatigue and response bias, and probably reducing the response rate. Therefore, Morean et al. revised SCS in 2014, forming a simplified version of seven items in two dimensions (Morean et al., 2014). At present, this version has been translated into many languages, which proves that its reliability and validity have cross-cultural consistency. The Cronbach's α of the simplified self-control scale in this study was 0.897 (T1), 0.928 (T2), and 0.894 (T3).

### 5.3 Mobile Phone Addiction Tendency Scale

The scale was compiled by Xiong et al. (2012) and was improved by referring to Young's Internet addiction symptom scale (Young, 1998). The measurement dimensions of the scale include abstinence symptoms, conspicuous behavior, mood changes, and social comfort. Questions 1, 4, 6, 8, 10, and 12 in the questionnaire were used to evaluate withdrawal symptoms (negative psychological and physiological reactions when there is no mobile phone activity). Questions 5, 9, 13, and 15 were used to evaluate salience behavior (the use of mobile phones occupies the center of thought and behavior). Except for questions 2, 7, and 16 belonging to social comfort, i.e., the effect of smartphones on individuals' social interaction, all other questions were related to mood changes, i.e., individuals' changes of mood due to the use of smartphones. In this research, the Cronbach's α of MPATS is 0.957 (T1), 0.951 (T2), and 0.954 (T3), respectively.

### 5.4 Statistical analyses

The data were input into Excel 2016, and the 8-digit number after the student number was used as the retrieval parameter. The function was used to import the three test data into SPSS26.0 analysis software after the corresponding data were completed. The validity and reliability of the measurement tools were investigated using exploratory factor analysis, confirmatory factor analysis, reliability analysis, and so on. After the data were standardized, descriptive statistics, the Mann-Whitney *U*-test, and other mathematical statistics were used to investigate the gender differences of each variable. The synchronous correlation and stable correlation of each variable are investigated by correlation analysis. AMOS25.0 software was used to construct the cross-lagged relationship model, and the maximum likelihood approach was employed to investigate the model fitting. The asynchronous correlation between variables was investigated from the time series through the cross-lagged path coefficient. At the same time, the relationship between variables was inferred according to the theoretical point of view of Eisma et al. (2019).

## 6 Results

### 6.1 Common method bias test

In this study, Harman's one-factor test method and the procedural control method were used to investigate the possible common method bias in the test. First, this study selected measurement tools repeatedly confirmed by domestic and foreign scholars to have high reliability and validity, and emphasized the anonymity and confidentiality of the survey in the questionnaire guide section. Second, after the basic and coding information were excluded, this study conducted an exploratory factor analysis on all items with unrotated single factors in the three tests. Eight factors with eigenvalues >1 were extracted from the three tests. The variation rates of the first factor were 39.885% (T1), 44.255% (T2), and 43.670% (T3), respectively, which did not reach the critical value of 50%. This indicates that the common method bias in the three tests was acceptable.

### 6.2 Gender differences in physical activity, self-control, and MPA

The Mann–Whitney *U*-test was performed on each variable of the three tests. The results showed that (Table 1) different gender samples did not show significant differences in T1 SC, T1 MPA, T2 MPA, and T3 MPA. In addition, the gender sample showed significant differences in T1 PA, T2 PA, T2 SC, T3 PA, and T3 SC. The PA of boys was better than that of girls, and the gender difference effect of PA was 0.209 (Cohen's *d* = 0.427), 0.118 (Cohen's *d* = 0.238), and 0.104 (Cohen's *d* = 0.210), respectively. The level of SC in T2 and T3 was higher in boys than in girls. The effect sizes of gender differences were 0.133 (Cohen's *d* = 0.268) and 0.114 (Cohen's *d* = 0.230), respectively.

**Table 1:** Mann–Whitney *U*-test of gender (M ± SD).

	**T1 PA**	**T1 SC**	**T1 MPA**	**T2 PA**	**T2 SC**	**T2 MPA**	**T3 PA**	**T3 SC**	**T3 MPA**
Mann-Whitney *U*	16,436.000	20,264.000	20,311.000	18,216.500	18,326.000	21,164.000	18,510.000	18,557.500	19,247.000
*Z*	−4.071	−0.914	−0.875	−2.603	−2.510	−0.173	−2.360	−2.320	−1.750
*P*	0.000^**^	0.361	0.382	0.009^**^	0.012^*^	0.863	0.018^*^	0.020^*^	0.080
Male	40.15 ± 31.99	24.35 ± 6.34	57.84 ± 13.99	37.67 ± 30.22	25.81 ± 6.69	55.86 ± 13.74	38.99 ± 30.53	27.27 ± 5.86	53.24 ± 14.81
Female	27.66 ± 26.22	24.89 ± 6.52	56.51 ± 14.45	30.75 ± 27.95	23.92 ± 7.42	55.68 ± 14.20	32.77 ± 28.72	25.90 ± 6.05	55.70 ± 14.71

^*^p < 0.05.

^**^p < 0.01.

### 6.3 Partial correlation analysis of physical activity, self-control, and MPA

Partial correlation analysis with gender differentiation was performed on each variable. First, we conducted stable correlation tests for the three variables, finding positive significance in each variable. Particularly, through the three tests on physical activities (PA), we found a significantly positive relationship between T1 PA and T2 PA (*r* = 0.499, *P* < 0.01), T1 PA and T3 PA (*r* = 0.118, *P* < 0.05), and T2 PA and T3 PA (*r* = 0.390, *P* < 0.01), respectively. Through the three tests on self-control (SC), we found positive significance between T1 SC and T2 SC (*r* = 0.481, *P* < 0.01), T1 SC and T3 SC (*r* = 0.248, *P* < 0.01), and T2 SC and T3 SC (*r* = 0.461, *P* < 0.01), respectively. Through the three tests on MPA, we found positive significance between T1 MPA and T2 MPA (*r* = 0.570, *P* < 0.01), T1 MPA and T3 MPA (*r* = 0.292, *P* < 0.01), and T2 MPA and T3 MPA (*r* = 0.620, *P* < 0.01). Second, we conducted synchronous correlation tests three times. Through the three tests, we found a consistently significant relationship between three groups of dimensions, i.e., between T1 PA, T1 SC, and T1 MPA, between T2 PA, T2 SC, and T2 MPA, and between T3 PA, T3 SC, and T3 MPA (Table 2). It shows that PA, SC, and MPA meet stable and synchronous correlations across 12 months, which is suitable for cross-lagged analysis.

**Table 2:** Partial correlation analysis of each variable.

**Variable**	**M**	**SD**	**T1 PA**	**T1 SC**	**T1 MPA**	**T2 PA**	**T2 SC**	**T2 MPA**	**T3 PA**	**T3 SC**	**T3 MPA**
T1 PA	33.604	29.740	1								
T1 SC	24.635	6.433	0.291^**^	1							
T1 MPA	57.145	14.230	−0.232^**^	−0.235^**^	1						
T2 PA	34.043	29.225	0.499^**^	0.216^**^	−0.117^*^	1					
T2 SC	24.821	7.138	0.514^**^	0.481^**^	−0.172^**^	0.488^**^	1				
T2 MPA	55.763	13.964	−0.386^**^	−0.511^**^	0.570^**^	−0.440^**^	−0.470^**^	1			
T3 PA	35.732	29.723	0.118^*^	0.070	−0.079	0.390^**^	0.330^**^	−0.271^**^	1		
T3 SC	26.553	5.995	0.328^**^	0.248^**^	−0.111^*^	0.576^**^	0.461^**^	−0.348^**^	0.358^**^	1	
T3 MPA	54.531	14.791	−0.495^**^	−0.413^**^	0.292^**^	−0.438^**^	−0.592^**^	0.620^**^	−0.311^**^	−0.511^**^	1

^*^p < 0.05.

^**^p < 0.01.

### 6.4 Cross-lagged analysis of physical activity, self-control, and MPA

**Figure 1:**
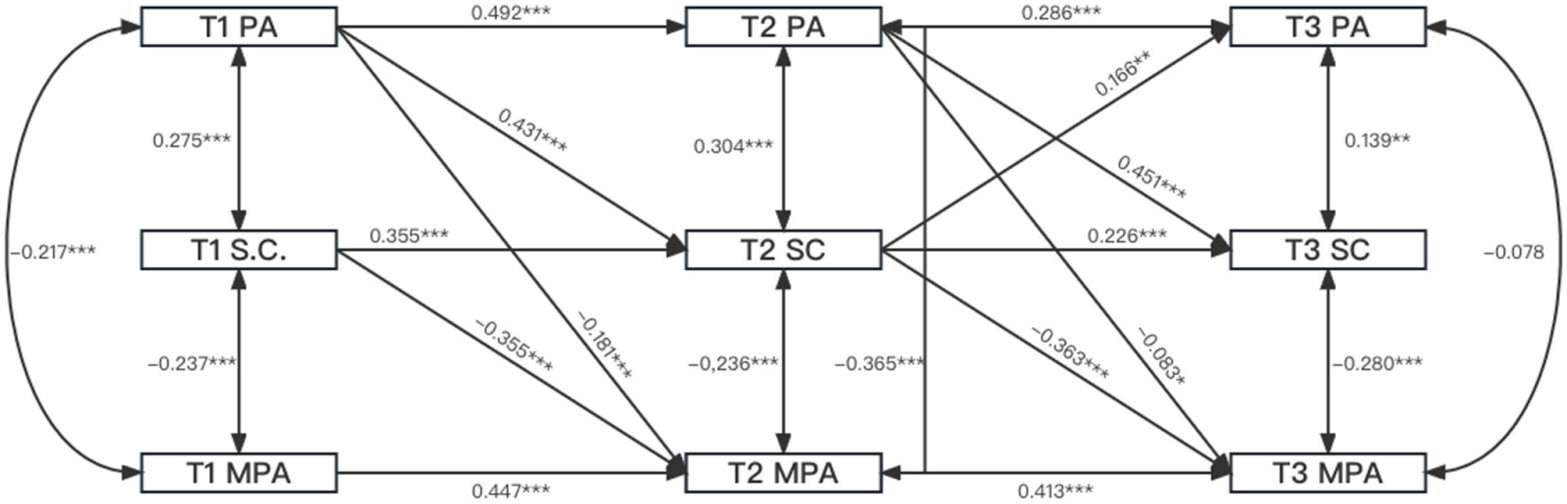
The cross-lagged effect model of physical activity, self-control, and MPA. ^*^: *p* < 0.05 (statistically significant). ^**^: *p* < 0.01 (highly statistically significant). ^***^: *p* < 0.001 (very highly statistically significant).

This study employed AMOS25.0 software to construct a cross-lagged effect model and used the maximum likelihood method to investigate the model fitting (Figure 1). The fitting indexes of the model showed: *x*^2^/df = 5.098, GFI = 0.977, NFI = 0.969, IFI = 0.975, CFI = 0.974; RMSEA = 0.100, SRMR = 0.030.

The asynchronous correlation between SC, MPA, and adolescent PA was investigated using the path coefficient of the cross-lagged effect model. (1) There was a significant correlation between T1PA and T2 SC (β = 0.431, *P* < 0.001) and between T1PA and T2 MPA (β = −0.181, *P* < 0.001). T1SC was significantly correlated to T2 MPA (β = −0.355, *P* < 0.001) but was not significantly correlated to T2PA (β = 0.077, *P* = 0.084). Besides, there was no significant correlation between T1MPA and T2SC (β = 0.014, *P* = 0.728) or between T1MPA and T2PA (β = 0.015, *P* = 0.742). (2) T2PA had a significant effect on T3SC (β = 0.451, *P* < 0.001) and T3MPA (β = −0.083, *P* < 0.05). There was a significant correlation between T2SC and T3MPA (β = −0.363, *P* < 0.001) and between T2SC and T3PA (β = 0.166, *P* < 0.05). There was no significant correlation between T2MPA and T3SC (β = −0.043, *P* = 0.339) or between T2MPA and T3PA (β = −0.067, *P* = 0.194; Figure 1). Based on the experience of variable relationship analysis with the aid of the cross-lagged model, the calculation conducted by combining the path coefficient of the model shows that PA and SC are the antecedent variables of MPA, and PA is the antecedent variable of SC. In addition, SC serves as a mediator in the path of PA, affecting MPA.

### 6.5 Analysis of the mediating effect of self-control

To test the mediating role of T2 SC in T1 PA and T3 MPA, this study conducted mediating effect testing and confidence interval estimation using the bias-corrected non-parametric percentile Bootstrap method. The specific effect value is shown in the Bootstrap mediating effect test, the confidence number is 2000, and the results are shown in Table 3.

**Table 3:** Tests the mediation effect of Bootstrap.

	**Effect**	**95% CI**		***z*/*t***	***P***	**Proportion**
		**LLCI**	**ULCI**			
T1 PA-T2 SC-T3 MPA	−0.120	−0.310	−0.182	−3.769	0.000	48.4%
T1 PA-T3 MPA	−0.128	−0.172	−0.085	−5.756	0.000	51.6%
Total	−0.249	−0.290	−0.207	−11.719	0.000	

From the Bootstrap mediation effect test in the above table, it can be observed that the direct effect of path T1 PA → T3 MPA is 0.128, with a 95% confidence interval of [−0.172, −0.085], excluding 0, which indicates a significant direct effect. For the path T1 PA → T2 SC → T3 MPA, the indirect effect is 0.120, with a 95% confidence interval of [−0.310, −0.182], also excluding 0, indicating a significant indirect effect. This indirect effect accounts for 48.4% of the total effect.

## 7 Discussion

### 7.1 Gender differences in physical activity, self-control, and MPA

First, men's level of physical activity is higher than women's and shows a stable gender difference across 12 months, and this finding shares similarities with former research (Yang et al., 2019; Buizza et al., 2022). This may be primarily related to men's higher interest in sports activities than women. As a symbol of strength and vitality, sports activities better cater to Chinese people's expectations of male personality traits. Under this influence, men are more physically active than women. Additionally, in the traditional Chinese gender concept, women are labeled as quiet and gentle, which leads most women to consciously reject high-intensity confrontational sports and choose low-intensity sports activities (walking and yoga). In contrast, men prefer to participate in high-intensity sports (basketball, football, and volleyball). Therefore, the gender differences in college students' sports activities may be related to the traditional Chinese gender concept and the sports preferences of men and women.

Second, MPA manifests gender consistency, which also shares similarities with former research (Khan et al., 2017). College students are interested in new things and are susceptible to MPA. Although college students exhibit distinctive traits and gender perceptions, they show relatively consistent gender differences in various aspects, such as lifestyles and attention preferences. For example, men mainly use mobile phones to play games or engage in other activities, while women are more likely to use mobile phones for social networking, browsing media information, and other social activities. However, the characteristics of disinhibition and editability in mobile phone use allow college students to avoid direct evaluation and reduce social anxiety moderately, thus obtaining consistent compensatory freshness and satisfaction (Lee et al., 2014). Moreover, due to the confounding effect caused by psychological and social factors related to MPA, college students may show gender identity in terms of frequency of use and psychological dependence. In addition, similar to previous research (Jiang and Zhao, 2016; Yang et al., 2019; Zhong et al., 2021), this study revealed higher SC levels in male college students at T2 and T3 compared to female students. This difference may be related to the levels of physical activity and MPA among the participants in this research.

### 7.2 The causal relationship between physical activity, self-control, and MPA

First, the cross-lagged model shows a delayed negative predictive effect of physical activity on MPA, enhancing previous research findings; this indicates that physical activity not only negatively affects the current level of MPA (Lepp et al., 2013; Kim et al., 2015; Towne et al., 2017) but also has a predictive effect on reducing subsequent levels of MPA. On the one hand, increased time spent on physical activities reduces the time available for mobile phone use (Wan and Ren, 2023). Active participation in sports helps decrease college students' use of the Internet and sedentary behavior (Kim and Sohn, 2014), thereby decreasing their mobile phone usage time and weakening their MPA. On the other hand, from a physiological perspective, engaging in physical activity shifts the excitement center of the cerebral cortex, allowing previously active nerve cells to rest and adjust. Therefore, when college students with MPA engage in physical activities, their attention shifts from mobile phones to exercise, which helps to alleviate their MPA levels.

Second, the cross-lagged model shows that physical activity can positively predict self-control in a delayed manner. In other words, college students with higher levels of physical activity develop stronger self-control after 6 months. These study results support and enrich previous theories and research results, suggesting that physical activity not only positively affects self-control but also has a lag effect. The delayed predictive effect of physical activity on self-control can be explained by the strength model of self-control. According to this model, the power consumption of self-control may be similar to the use of muscles, which can become strong through regular activities or recover through appropriate rest after a period of exercise (Muraven and Baumeister, 2000). In sports, especially endurance events (marathons, cycling races, and swimming), people often feel tempted to give up when facing physical and psychological challenges. However, overcoming this impulse and persisting enhances self-control abilities (Oaten and Cheng, 2006). College students inevitably consume energy during physical activities. If they can control themselves to complete their activity plans, their self-control abilities improve, albeit with a potential lag. Therefore, college students can effectively improve their self-control abilities by frequently participating in physical activities and developing persistence and enthusiasm for physical activities.

Third, the cross-lagged model shows that self-control can negatively predict college students' MPA within 12 months in a delayed manner. This means students with higher self-control have lower MPA levels after 6 months. This finding further validates previous studies to a certain extent (Kim and Sohn, 2014; Jiang and Zhao, 2016), and aligns with the addictive behavior model (Brand et al., 2016). The occurrence of any behavior is driven by motivation to meet certain psychological needs. Additionally, according to the self-determination theory, the healthy development of individuals depends highly on whether their basic psychological needs, i.e., competence, autonomy, and belonging, can be met. People with stronger self-control can modify their behavior and performance to meet societal needs.

Self-control is a process of self-selection and continuous efforts to improve oneself, which helps individuals foster a stronger sense of autonomy. These people tend to develop higher workability and relatively easily foster a sense of competence. Additionally, their behavior can better meet social expectations and is more easily recognized and appreciated by others, which improves their sense of belonging. Therefore, people with stronger self-control abilities can better satisfy their basic psychological needs and are less likely to develop unhealthy addictive behaviors. On the other hand, according to the ACE model of Internet addiction (Young and Rogers, 1998), mobile phone networks possess characteristics of anonymity, convenience, and escape. Anonymity allows individuals to fabricate false information to create false personas and hide their real goals and information. Convenience refers to cyberspace shortening communication distances and enabling zero-distance interaction, with relatively simple interactive operations providing great convenience for users. Escape refers to individuals who cannot withstand the impact of reality and challenges, escaping to the virtual network world to avoid reality, paralyze themselves with online pleasure, and escape from real problems. For college students with low self-control, the above characteristics have become a means to fulfill psychological needs, resulting in mobile phone addiction. Therefore, improving self-control is undoubtedly an effective and feasible way for college students to deal with MPA.

Finally, apart from the delayed predictive effect discussed above, the cross-lagged model tests also observed a partial mediating role of self-control across the three-time points in the impact of physical activity on MPA. In other words, T1 physical activity may indirectly affect T3 MPA via T2 self-control. At the same time, T1 physical activity may also directly affect T3 MPA. The research findings support and enrich former studies and literature (Yang et al., 2019; Zhong et al., 2021), which reveal the mechanisms of physical activities and self-control at the vertical level to some extent. Physical activity and self-control can not only alleviate the current MPA level but also generate a certain lag and delay effect. This means that the exploration of current college students' MPA cannot be limited to current influencing factors; it must also consider the role of factors from a previous period. Given that physical activity can directly and indirectly influence MPA, the relationship between physical activity and MPA may be complex. The direct impact of physical activity on MPA can be explained and proven by the previous studies discussed above (Lepp et al., 2013; Kim et al., 2015; Towne et al., 2017). The indirect effect of physical activity on MPA can be explained by the self-control power model: physical activity that persists for a certain period of time may trigger a higher level of self-control, but the stronger self-control ability may not immediately affect MPA due to its lag effect. Individuals with stronger self-control are more likely to participate in healthy activities that bring long-term benefits and satisfaction (Jiang and Zhao, 2016). Then, the pleasure and satisfaction gained from physical activities drive them to use mobile phones less, thus reducing MPA.

### 7.3 Limitations

Although this study reveals the impact mechanisms of physical activity, self-control, and MPA among college students at the vertical level, there are still some shortcomings. First, the study focuses only on students from a university in Hunan Province, China, limiting the sample's representation and the generalization of the results. Second, this study relies on subjective self-report questionnaires. Future research can use artificial intelligence devices, such as pedometers and sports bracelets, to measure physical activity to obtain more accurate and quantitative data, such as walking steps, heart rate, and blood pressure. Finally, in addition to self-control, other important psychological indicators may also impact the association between physical activity and MPA. Therefore, such variables should be explored in future research.

## 8 Conclusion

This study investigated 414 college students and observed remarkable gender differences in physical activity, self-control, and MPA. According to the longitudinal data analysis, physical activity has a direct and significant impact on MPA levels. In particular, college students who engage in more physical activities are less dependent on mobile phones. Besides, physical activity can affect subsequent self-control levels. College students who participate in more physical activities exhibit stronger self-control abilities. Finally, self-control has a delayed impact on MPA levels, with students who have stronger self-control being less dependent on mobile phones. Moreover, this study also finds that physical activity can indirectly affect MPA levels through self-control. Overall, this study suggests that issues related to individual mobile phone addiction should be viewed from a developmental perspective.

## Data availability statement

The original contributions presented in the study are included in the article/supplementary material, further inquiries can be directed to the corresponding author.

## Ethics statement

The studies involving humans were approved by the Ethics Committee of the School of Physical Education, Hunan University of Science and Technology. The studies were conducted in accordance with the local legislation and institutional requirements. Written informed consent for participation in this study was provided by the participants' and the participants' legal guardians/next of kin.

## Author contributions

QW: Data curation, Methodology, Writing – original draft, Writing – review & editing. YC: Data curation, Formal analysis, Writing – review & editing. LL: Data curation, Formal analysis, Methodology, Supervision, Validation, Writing – review & editing.
